# New Resources for the Specific and Sensitive Detection of the Emerging Tomato Brown Rugose Fruit Virus

**DOI:** 10.3390/v13091680

**Published:** 2021-08-25

**Authors:** Joan Miquel Bernabé-Orts, Covadonga Torre, Eduardo Méndez-López, Yolanda Hernando, Miguel A. Aranda

**Affiliations:** 1 Abiopep S.L. Parque Científico de Murcia. Ctra. Madrid Km 388, Complejo Espinardo. Edificio R 2ª Planta, Espinardo, 30100 Murcia, Spain; jm.bernabe@abiopep.com (J.M.B.-O.); ctorre@abiopep.com (C.T.); yh.saiz@abiopep.com (Y.H.); 2 Centro de Edafología y Biología Aplicada del Segura, Consejo Superior de Investigaciones Científicas (CSIC), Campus Universitario de Espinardo, Edificio 25, Espinardo, 30100 Murcia, Spain; fmendez@cebas.csic.es

**Keywords:** ToBRFV, RT-qPCR, LAMP, DAS-ELISA, plant virus, tobamovirus, detection

## Abstract

Plant viruses can evolve towards new pathogenic entities that may eventually cause outbreaks and become epidemics or even pandemics. Seven years ago, tomato brown rugose fruit virus (ToBRFV) emerged, overcoming the genetic resistance that had been employed for more than sixty years against tobamoviruses in tomato. Since then, ToBRFV has spread worldwide, producing significant losses in tomato crops. While new resistances are deployed, the only means of control is the implementation of effective prevention and eradication strategies. For this purpose, in this work, we have designed, assessed, and compared an array of tests for the specific and sensitive detection of the ToBRFV in leaf samples. First, two monoclonal antibodies were generated against a singular peptide of the ToBRFV coat protein; antibodies were utilized to devise a double-antibody-sandwich enzyme-linked immunosorbent assay (DAS-ELISA) test that sensitively detects this virus and has no cross-reactivity with other related tobamoviruses. Second, a real-time quantitative PCR (RT-qPCR) test targeting the RNA-dependent replicase open reading frame (ORF) was designed, and its performance and specificity validated in comparison with the CaTa28 and CSP1325 tests recommended by plant protection authorities in Europe. Third, in line with the tendency to use field-deployable diagnostic techniques, we developed and tested two sets of loop-mediated isothermal amplification (LAMP) primers to double-check the detection of the movement protein ORF of ToBRFV, and one set that works as an internal control. Finally, we compared all of these methods by employing a collection of samples with different ToBRFV loads to evaluate the overall performance of each test.

## 1. Introduction

**Figure 1:**
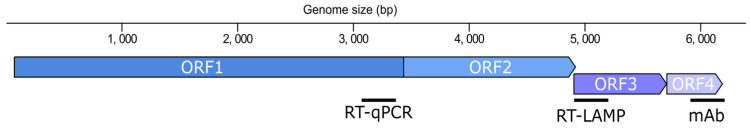
Genome organization of ToBRFV and regions targeted by the different tests (RT-qPCR, RT-LAMP, and ELISA). RT-qPCR was directed to the 3′ end of the large fragment of the *RdRP* (ORF1), RT-LAMP to the start of the *MP* (ORF3), and ELISA against the C-terminus of the CP, which is the gene product of ORF4.

Global food security is constantly threatened by emerging pathogens, such as plant viruses. Plant viruses cause outbreaks that reduce the yield and the quality of the crops that sustain the food chain [1]. The globalization of the vegetable trade, together with the limited effectiveness of monitoring and eradication measures, favor the worldwide spread of plant viruses. In addition, the frequency and scale of plant virus outbreaks are predicted to increase due to climate change, which may facilitate the expansion of new or existing viruses to geographic areas in which they were not previously present or epidemic [2]. This is the case of tomato brown rugose fruit virus (ToBRFV) (genus *Tobamovirus*, family *Virgaviridae)*. ToBRFV, as other tobamoviruses, has a particle with a rod-shaped morphology formed by multimers of the capsid protein (CP) that encapsulate a positive single-stranded RNA (ssRNA(+)) genome of 6.2 to 6.4 kb encoding four open reading frames (ORFs) (Figure 1). ORF1 and ORF2 are separated by a leaky stop codon and encode non-structural proteins that assemble the RNA-dependent RNA polymerase (RdRP). ORF1 encodes the small subunit (124–132 kDa) and ORF2 the large subunit of the RdRP (181–189 kDa) which contains the polymerase domain. The downstream ORFs encode the 28–31 kDa movement protein (MP) and the 17–18 kDa CP, which are translated from their respective subgenomic RNAs. 

The tobamovirus group includes well-known viruses that affect tomato and other solanaceous species, such as tobacco mosaic virus (TMV) and tomato mosaic virus (ToMV) among others [3,4]. Thus far, the *Tm-1, Tm-2*/*Tm-2^2^* resistance genes have routinely been used in tomato hybrid breeding to protect new varieties against TMV and ToMV [5,6]. However, the *Tm-2^2^* genetic resistance which lasted unbroken for over sixty years has now been overcome by ToBRFV. Apparently, the MP of ToBRFV is the genetic determinant for *Tm-2^2^* resistance-breaking [7]. Similarly, the *L* gene alleles have been used for the protection of most pepper commercial cultivars against tobamoviruses. ToBRFV induces a hypersensitive response in plants harboring these resistance genes [8], and infections of sweet pepper lacking these resistances have been reported in Italy [9]. On the other hand, the viral particle of ToBRFV is very stable and resilient to commonly used disinfectants [10], which may facilitate its mechanical transmission even by pollinators [11]. Taken together, the particular features of ToBRFV have contributed to its rapid spread worldwide [12,13,14,15,16,17,18,19,20,21] since it was first reported in Israel and Jordan in 2014 [8,22]. In an attempt to delay or arrest ToBRFV expansion in Europe, it has been included in the European Plant Protection Organization (EPPO) alert list (Commission Implementing Decision EU 2019/1615) and in the list of quarantine bodies (Commission regulation EU 2019/2072). 

Detection of infected plants or seeds is critical for the success of the intervention and eradication strategies to prevent further expansion of ToBRFV. In this matter, the EPPO published a Standard that describes a diagnostic protocol for the detection and identification of this virus (PM7/146(1)). This protocol distinguishes between plant and seed materials. Thus, symptomatic plant material can be processed by enzyme-linked immunosorbent assay (ELISA), while the recommendation for asymptomatic material is the analysis by coupled reverse transcription-polymerase chain reaction (RT-PCR) and real-time quantitative PCR (RT-qPCR). In contrast, seed material must be exclusively analyzed by RT-qPCR. The RT-PCR should be performed using primers from Alkowni et al., 2019 [19] or Rodriguez-Mendoza et al., 2019 [23], while the RT-qPCR should be carried out by using a duplex real-time test with CaTa28 and CSP1325 primers and probe proposed by the International Seed Federation (ISF) in 2020. Apart from the EPPO’s Standards, other RT-qPCR tests have been developed for ToBRFV detection and compared with RT-PCR tests [24]. As an alternative to these tests, isothermal amplification techniques such as loop-mediated isothermal amplification (LAMP) [25] are gaining popularity due to their simplicity and similar performance compared to RT-qPCR. Many tests have been developed using LAMP to diagnose plant viruses, including ToBRFV [26,27]. 

In spite of the existence of a handful of resources for the detection of ToBRFV, a comprehensive comparison that facilitates their selection is still missing. In this work, we have designed, assessed, and compared new tests for the specific and sensitive detection of the ToBRFV viral particle and its genome. To this end, we have targeted the CP and two different ORFs (*RdRP* and *MP*) (Figure 1) by employing three different methods: DAS-ELISA, RT-qPCR, and RT-LAMP. Altogether, we provide an array of new resources for ToBRFV testing at the protein and nucleic acid levels that are specific to this virus and show an equivalent sensitivity to those of the EPPO-recommended tests.

## 2. Materials and Methods

### 2.1. Virus Isolates and Plant Inoculation

The Spanish ToBRFV isolate from Vicar (Almeria, Spain) [18] was provided by the “Laboratorio de Producción y Sanidad Vegetal” (La Mojonera, Almería, Spain). Isolates of other tobamoviruses used in this study were acquired from the DSMZ (Leibniz, Germany) collection: TMV (PV-1252), ToMV (PV-0141), pepper mild mottle virus (PMMoV, PV-0093), tobacco mild green mottle virus (TMGMV, PV-0124). Approximately 50 mg of dried plant tissue was homogenized for each isolate in 2 mL of 30 mM phosphate buffer pH = 8 using a mortar and pestle. Homogenates were used to mechanically inoculate leaves of 25–26-day-old *N. benthamiana* plants (4–5-true leaves) and the first pair of true leaves of 7–10-day-old tomato plants (cultivar M82). For this, the leaves to be inoculated were first dusted with carborundum powder (600 mesh) and then manually rubbed with the homogenate. The plants thus inoculated were kept separately in a confined greenhouse under controlled conditions set with a 16/8 h photoperiod and a 26/22 °C day/night cycle. After 10 to 15 days, systemic leaves that showed obvious symptoms of infection were collected, cut, mixed, and divided into samples of approximately 100 mg. The samples were frozen in liquid nitrogen, ground with a Retsch Mixer Mill MM400 (ThermoFisher, Hampton, NH, USA) for 1 min at 30 Hz, and stored at −80 °C for later analyses.

### 2.2. Design and Production of Monoclonal Antibodies against ToBRFV

**Figure 2:**
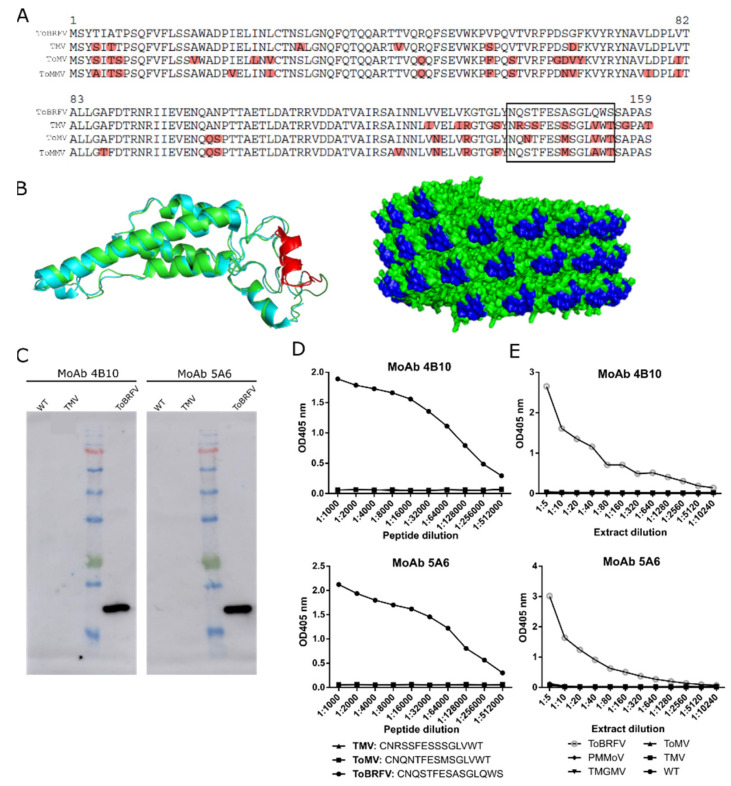
Design and evaluation of two monoclonal antibodies (MoAb 4B10 and MoAb 5A6) against ToBRFV CP. (**A**) Clustal alignment of the CP polypeptide sequences from ToBRFV (NC_028478), TMV (NC_001367), ToMV (NC_002692), and ToMMV (NC_022230) for the identification of a ToBRFV peptide candidate to give rise to specific ToBRFV antibodies (squared is the best candidate). (**B**) On the left, ribbon representation of the structural superposition of the TMV (blue) and the predicted ToBRFV CPs (green). The selected peptide is highlighted in red. On the right, molecular surface representation of the assembled virion particle of TMV with the selected peptide marked in blue. The TMV CP structure was obtained from the protein data bank (2om3, [33]) and the ToBRFV CP sequence from Uniprot (A0A0S2SZX3, [34]). Protein structure visualization was performed with Pymol. (**C**) Western blot analyzing the exclusivity of MoAb 4B10 and MoAb 5A6. Extracts from healthy (WT), TMV- and ToBRFV-infected plants were loaded. (**D**) Indirect ELISA results showing the exclusivity of MoAb4B10 or 5A6 when using serial dilutions of synthetic peptides (squared in (A)) for ToBRFV, ToMV, and TMV. (**E**) Analytical sensitivity and exclusivity of the DAS-ELISA tests devised with the monoclonal antibodies. Each data point represents a single measurement.

The monoclonal antibodies (MoAbs) were produced by GenScript (Piscataway, NJ, USA) using standard methods [28,29]. Briefly, the ToBRFV CP epitope (Figure 2) was synthesized and used as an immunogen to be injected into five 6-week-old BALB/c mice. Production of hybridoma-secreting MoAbs against the ToBRFV was performed according to [30] with minor modifications. Hybridoma supernatants were screened for the presence of anti-CP peptide antibodies by indirect ELISA in 96-well plates and Western-blotting. Positive hybridoma clones were cultured in Dulbecco’s Modified Eagle’s Medium (DMEM) high glucose (WISENT Bioproducts, Quebec, Canada), supplemented with 10% fetal bovine serum (WISENT Bioproducts) and 1% Penicillin-Streptomycin (Sigma-Aldrich, Saint Louis, MO, USA). Cells were allowed to grow at 37 °C and supplemented with 5.5% of CO_2_. The hybridoma was then injected intraperitoneally into pristine primed syngeneic BALB/c mice to produce ascites. After 7–10 days, the ascites samples were collected and their titers were determined by indirect ELISA. The isotypes of the MoAbs were determined using an isotyping kit following the manufacturer’s instructions (Sigma-Aldrich). The anti-CP peptide IgG was purified from ascites with an immobilized protein-G affinity column (GE Healthcare, Wauwatosa, WI, USA) according to the manufacturer’s manual. Purified antibodies were stored at −80 °C.

### 2.3. Western Blot

Healthy and ToBRFV-, TMV- or ToMV-infected tomato leaves were ground in liquid nitrogen using a mortar and a pestle. The plant material was solubilized in 4 mL per g of RIPA buffer (10 mM Tris-HCl pH = 7.5; 150 mM NaCl; 0.5 mM EDTA; 0.1% SDS; 1% Triton X-100; 1% Deoxycholate) and mixed thoroughly. The homogenate was centrifuged at 3000× *g* for 15 min at 4 °C, then the supernatant was transferred to a new tube and centrifuged at 12,000× *g* for 15 min at 4 °C. The extracts were kept at −20 °C. The proteins were resolved in 15% SDS-PAGE gels and blotted onto nitrocellulose membranes (GE Healthcare) using a Trans-Blot (Bio-Rad, Hercules, CA, USA). After blocking, the membranes were incubated with a 1/250 dilution of antisera from immunized mice against the ToBRFV CP peptide or with 1/2 hybridoma supernatant dilutions. Next, an HRP-conjugated anti-mouse secondary antibody (Promega, Madison, WI, USA) was used at a 1/2500 dilution. As a control, a commercial ToBRFV antibody (DSMZ, Leibniz, Germany) was used at 1/1000 dilution and detected with an HRP-conjugated anti-rabbit secondary antibody (Promega) at 1/2500 dilution. The membranes were developed using SuperSignal West Pico PLUS (ThermoFisher, Hampton, NH, USA) and an Amersham Imager 600 (GE Healthcare Life Sciences, Wauwatosa, WI, USA).

### 2.4. DAS-ELISA

Plant extracts were obtained by homogenizing ground tissue with extraction buffer (137 mM NaCl, 2.7 mM KCl, 10 mM Na_2_HPO_4_, 1.8 mM KH_2_PO_4_, 0.05% Tween-20, 20 g/L polyvinyl pyrrolidone (K10-K40), 2g/L bovine serum albumin, pH = 7.4) in a 1:5 ratio (μg: mL). The homogenate was centrifuged for 10 min at 13,000 rpm. The supernatant was recovered and used in the ELISA as follows. A 96-well plate (ThermoFisher) was coated with 50 μL/well of the capture antibody diluted in coating buffer (15 mM Na_2_CO_3_, 35 mM NaCO_3_, pH = 9.6) and incubated for 3 h at 37 °C. This was followed by four washes with 200 μL/well of wash buffer (137 mM NaCl, 2.7 mM KCl, 10 mM Na_2_HPO_4_, 1.8 mM KH_2_PO_4_, 0.05% Tween-20, pH = 7.4). Afterwards, 50 μL/well of the plant extract were added and incubated overnight at 4 °C. Once washed four times with 200 uL/well of wash buffer, 50 μL/well of the secondary antibody (tagged with the alkaline phosphatase reporter enzyme) was added, and the plate incubated for 3 h at 37 °C, after which the washing step was repeated as above. Lastly, the enzymatic activity was measured after adding 50 μL of the substrate solution (1 mg/mL 4-nitrophenylphosphate-di-Na-salt in substrate buffer (1 M diethanolamine, pH = 9.8) and incubating the plate for 60 min, then the absorbance was read at 405 nm using a TECAN Sunrise (Männedorf, Switzerland). The AbCam Alkaline phosphatase Conjugation Kit—Lightning-Link^®^ (AbCam, Cambridge, UK) was used to conjugate the secondary antibody following the manufacturer´s instructions. 

### 2.5. RNA Extraction

RNA extraction was performed using the NucleoSpin RNA plant kit (MACHEREY-NAGEL, Düren, Germany) following the manufacturer’s instructions. The RNA extracts were checked by electrophoresis in a 1% agarose gel, and the RNA concentration was measured with a Nanodrop ™ One (ThermoFisher), and adjusted to a working concentration of 10 ng/µL, to be used as a template for the RT-LAMP and RT-qPCR. A healthy plant RNA extract was used as the diluent to adjust the concentrations.

### 2.6. Primers and Probe Design for One-Step RT-qPCR

A total of 71 complete sequences for ToBRFV, 31 complete sequences for the ToMV, 40 complete sequences for TMV, 16 complete sequences for PMMoV and 26 partial sequences for TMGMV were retrieved from the NCBI database to assess inclusivity for each RT-qPCR test. Sequence alignments were performed with the ClustalW tool [31] included in the MEGA7 software [32]. For RT-qPCR tests, a 122 bp region within the *RdRP* was chosen for ToBRFV, a 152 bp region of the *RdRP* for ToMV, a 148 bp of the *RdRP* for TMV, a 144 bp region of the *CP* for PMMoV, and a 106 bp segment of the *CP* for TMGMV. The Primer3 plus program was used to design the primers and probe set, and the theoretical properties of the designed assay were analyzed with the Oligo Analyzer tool from IDT (Integrated DNA Technologies, v3.1.1). The primers and probe exclusivity were also evaluated in silico against the other tobamoviruses known to affect tomato plants. Both the primers and probe were synthesized by IDT (Newark, NJ, USA) (Table S2).

### 2.7. In Vitro Transcription and Standard Curve

A synthetic fragment containing part of the ToBRFV ORF1 was synthetized by GenScript (Piscataway, NJ, USA) and cloned in a pBluescript II KS (+) vector (Stratagene, La Jolla, CA, USA). In vitro transcription was performed with T3 polymerase (Promega, Madison, WI, USA) in a 20 μL reaction according to the manufacturer’s instructions. After ethanol precipitation, the transcripts were resuspended in 20 μL of RNase-free water and the RNA concentration was measured spectrophotometrically with a Nanodrop ™ One (ThermoFisher). The standard curve was prepared from serial dilutions of the in vitro transcript in a 1:10 range, diluted in healthy tomato RNA extracts to a final concentration of 16 ng/μL.

### 2.8. One-Step RT-qPCR

The one-step RT-qPCR reactions were performed using the KAPA PROBE FAST Universal One-Step qRT-PCR kit (KAPA Biosystems, Wilmington, MA, USA) following the manufacturer’s instructions. Two microliters of nucleic acid preparation were added to a final volume of 10 μL. The reactions were carried out in a StepOne Plus ™ Real-Time PCR System (Applied Biosystems, Waltham, MA, USA) thermocycler, and the reaction conditions were a reverse transcription step of 5 min at 42 °C, followed by PCR with a 3 min denaturation cycle at 95 °C and 40 amplification cycles of 3 s at 95 °C, and 30 s at 60 °C. Three replicates were analyzed per plot/sample and two negative controls were included in each experiment, which consisted of RNA extracts of healthy tomato leaf diluted to a final concentration of 30 ng/μL, and water in the non-template control (NTC).

### 2.9. RT-LAMP

LAMP primers were designed using PrimerExplorer v.5 (https://primerexplorer.jp/e/, accessed on 22 May 2020). Two sets of primers against the *MP* of ToBRFV were designed (MP1 and MP2, Table S2). An additional set of primers targeting the tomato ribosomal RNA 25S subunit was also designed as an internal control (rRNA 25S, Table S2). RT-LAMP reactions were performed using the WarmStart LAMP Kit (NEB, Ipswich, MA, USA) at a final volume of 10 μL. Primers were added at a final concentration of 0.2 μM for F3 and B3, 1.6 μM for FIP and BIP primers, and 0.8 μM for LF and LB primers. Reactions were performed independently for each set of primers (MP1, MP2, and rRNA 25S) using 2 μL of input RNA. The amplification was performed at a constant temperature of 62 °C for 25–30 min (50–60 cycles of 30 s each) in an Applied Biosystems StepOnePlus Real-Time PCR System (Waltham, MA, USA), and tracked with a DNA-intercalating green fluorophore provided with the WarmStart LAMP Kit (NEB). 

### 2.10. ToBRFV Timecourse Experiment

In total, twenty-one 5-week-old tomato plants cv. M82 (3–4 pairs of leaves approximately) were mechanically inoculated (see above). Three plants per data point (1, 2, 3, 4, 6, 8, 12 days post-inoculation) were sampled, collecting the first pair of newly emerged leaves in which the virus has replicated systemically. Then, samples were finely sliced and two subsamples prepared, one containing 100 mg for RNA extraction, and another with 200 mg for protein extraction. These subsamples were frozen in liquid nitrogen, ground with a Retsch Mixer Mill MM400 (ThermoFisher) for 1 min at 30 Hz, and stored at −80 °C for later analyses.

## 3. Results

### 3.1. Development of Monoclonal Antibodies and Two DAS-ELISA Tests to Detect ToBRFV

Given the relatedness of the ToBRFV CP to other tobamoviral CPs (Figure 2A), we aimed at producing MoAbs able to recognize a specific ToBRFV CP epitope. 

To this end, we first identified small peptides within the ToBRFV CP which harbored dissimilarities with ToMV, TMV and ToMMV CP sequences. We next examined the location of dissimilar peptides in the tobamoviral CP structure. One of the selected peptides (squared in Figure 2A and shown in red in Figure 2B) had an α-helix shape, which may be beneficial for its antibody recognition. In addition, the selected peptide (shown in deep blue in Figure 2B) was displayed on the surface of the virion particle. Altogether, these data suggested that this peptide was a good candidate for the production of monoclonal antibodies exclusive to ToBRFV. Inclusivity of this immunization epitope was also corroborated aligning CPs of different ToBRFV isolates (Figure S3). After peptide expression, mice immunization, antisera immunogenicity characterization, and hybridoma production and selection (Figure S1), we used two hybridoma lines to produce MoAbs, herein referred to as MoAb 4B10 and MoAb 5A6. The exclusivity of these MoAbs was evaluated by Western blotting (Figure 2C) and ELISA (Figure 2D). Western blot results using protein extracts from tomato plants infected with TMV or ToBRFV showed that both antibodies were exclusive to ToBRFV. The same was found when the antibodies were subjected to indirect ELISA, coating the plate with serial dilutions of TMV, ToMV or ToBRFV synthetic peptides; only the ToBRFV peptide was recognized by both antibodies. We next focused on developing a DAS-ELISA test with MoAb 4B10 or 5A6. In both cases, the same antibody was used for capturing and detecting the antigen, with the only difference being the alkaline phosphatase conjugated to the detection antibody. Once the best concentrations of primary and secondary antibodies were defined for each pair of antibodies, the resulting combinations were used in a DAS-ELISA test (Figure 2E). Serial dilutions of protein extracts from infected *N. benthamiana* and tomato plants were used to assess the analytical sensitivity and exclusivity of the antibodies. The results indicated that only the extract from the ToBRFV-infected plants produced detectable signals for both MoAbs confirming their exclusivity for this virus. The tests with both MoAbs exhibited very low background levels, with cut-off values estimated at 0.055 and 0.086 for MoAb 4B10 and 5A6, respectively. ToBRFV was still detected at 1:10,240 dilution when using 4B10 antibody, while 5A6 antibody allowed ToBRFV detection up to a 1:5120 dilution, delimitating the analytical sensitivity of both tests. In summary, we produced a pair of monoclonal antibodies that could be used in specific and sensitive DAS-ELISA tests, Western-blot and possibly other applications.

### 3.2. Development of an RT-qPCR Test to Detect the RdRP of ToBRFV

**Figure 3:**
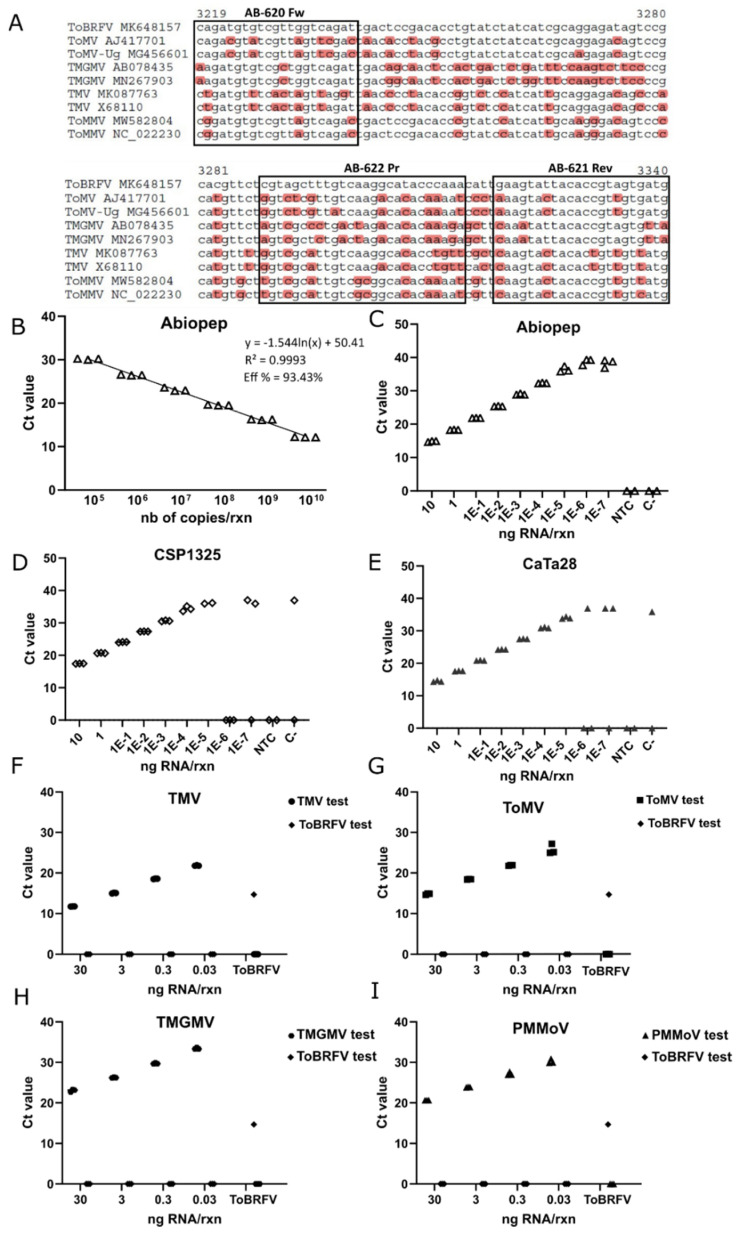
Design and evaluation of an RT-qPCR test for the detection of the ToBRFV *RdRP*. (**A**) Alignment of the large fragment of the ToBRFV *RdRP* 3′ end and other closely related tobamoviruses to examine the exclusivity of the oligonucleotides. Mismatches are shown in red and the selected oligonucleotides (AB-620 Fwd, AB-621 Rev, and AB-622 Pr) are indicated with rectangles. We have given the name of “Abiopep” to this set of oligonucleotides. The NCBI accession numbers for each virus sequence are shown together with the virus acronym. (**B**) Assessment of the Abiopep test sensitivity using serial dilutions of a synthetic transcript of an *RdRP* fragment. The Ct values versus the number of copies per reaction are plotted. (**C**–**E**) Evaluation of the analytical sensitivity of Abiopep, CaTa28 and CSP1325 tests using serial dilutions of total RNA extracted from tomato leaves infected with ToBRFV. (**F**–**I**) Analytical specificity of the Abiopep test checked using dilutions of RNA extracted from infected *N. benthamiana* leaves with related tobamoviruses. Each RNA extract was assessed with both a specific set of oligonucleotides designed for the amplification of that virus in particular (TMV (F), ToMV (G), TMGMV (H), or PMMoV(I)), and with the Abiopep test. As a positive control, ToBRFV RNA was used. For all the graphs, Ct values from three technical replicates are represented versus the nanograms of RNA per reaction (rxn). NTC and healthy plant RNA extract (WT) were used as negative controls. All the dilutions were made using a healthy plant RNA extract as diluent. Experiments in (B) and (C) were repeated three times.

Next, we sought to develop a new set of primers and probe that could be able to complement the existing CaTa28 and CSP1325 set. Thus, we designed the two specific oligonucleotides AB-620 Fw and AB-621 Rev for the exclusive and inclusive amplification of a 144 pb segment of the ToBRFV ORF1 from different isolates of this virus, and the TaqMan probe AB-622 Pr for its detection of the same (Figure 3A and Figure S3).

We named the new primers and probe set as “Abiopep”. Oligonucleotides were first assessed using serial dilutions of an internal control (IC, a synthetic RNA fragment encoding part of ORF1), showing an estimated amplification efficiency of 93% (Figure 3B). We further examined Abiopep´s analytical sensitivity using serial dilutions of an RNA extract from ToBRFV-infected tomato plants (Figure 3C) and compared it with the CSP1325 (Figure 3D) and the CaTa28 (Figure 3E) sets (see Table S1 for detailed values for each replicate). The three tests showed different Ct values throughout the range of RNA dilutions analyzed. In general, CaTa28 and Abiopep detected ToBRFV at lower Cts than CSP1325. At a 1E-5 ng/reaction (rxn) dilution, CSP1325 failed to detect one technical replicate. The same took place with CaTa28 but in the next dilution (1E-6 ng/rxn). In contrast, Abiopep still showed signals, reaching a plateau at Ct values beyond 1E-5 ng/rxn. This plateau was also observed for CaTa28 and CSP1325. To rationally define a Ct cut-off value to our test, we performed a meta-analysis using all the data generated by our diagnostic service during 2020. Thus, out of the 214 negative samples analyzed (WT and NTC), the 85 which showed detectable signals were considered (Figure S2). We found that almost all of the samples grouped on a median Ct value of approximately 38. Only two of these samples displayed a Ct value very close to 35 (34.7 and 35.9, respectively). Altogether, these data suggested that under our particular conditions of equipment, materials, and reagents, the Ct cut-off value may be set at a Ct <35. On the other hand, no cross-reactivity was detected when testing four related tobamoviruses. TMV, ToMV, TMGMV, and PMMoV, were included in the assay (Figure 3F–I). In these experiments, we used four dilutions of the RNA extracts from *N. benthamiana* plants infected with each virus and a sample infected with ToBRFV. All the samples were analyzed with both a specific set of primers and probe to the virus of interest (TMV, ToMV, TMGMV, or PMMoV), and Abiopep to detect ToBRFV. In every case, the detection of the target virus was only observed when using its corresponding set of primers and probe. The ToBRFV detection signal was only observed for the ToBRFV-infected sample, but not in the samples infected with the other viruses. 

### 3.3. Development of a LAMP Test to Detect the ToBRFV MP 

**Figure 4:**
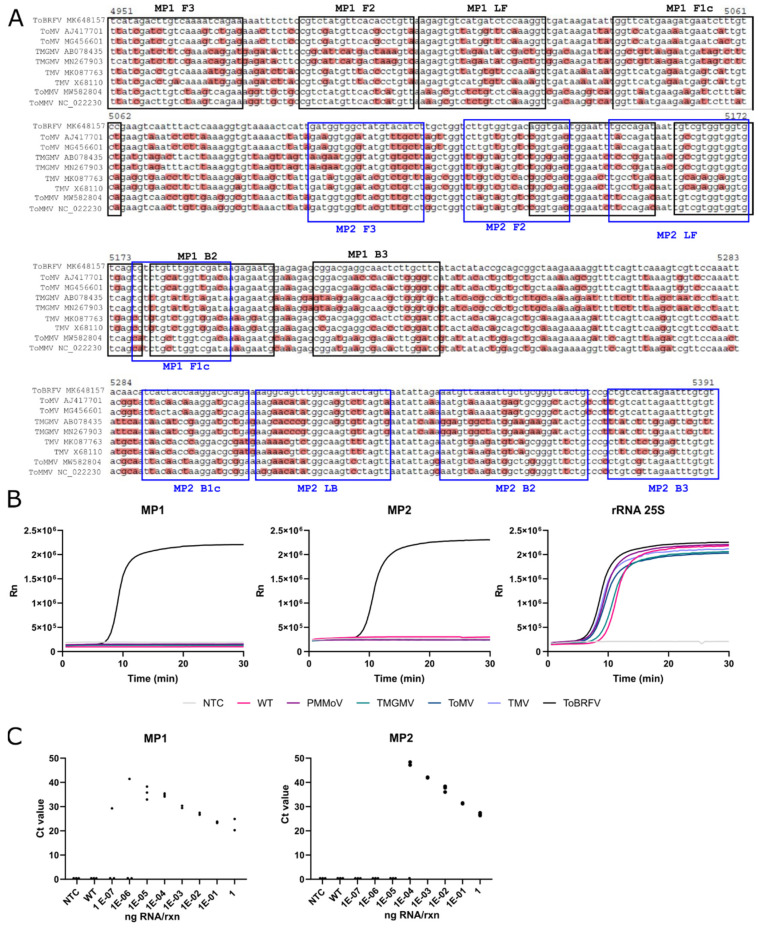
Design and evaluation of an RT-LAMP test for the detection of the ToBRFV *MP*. (**A**) Two sets of six oligonucleotides each (F3, B3, FIP, BIP, LF, and LB) named MP1 and MP2, were designed for the amplification of two segments of the ToBRFV *MP*. Black rectangles show the MP1 set and blue rectangles the MP2 oligonucleotides. An additional set of primers was designed to amplify the ribosomal RNA (rRNA) 25S (not shown) as an IC. (**B**) Assessment of the analytical specificity of all the LAMP primer sets using total RNA extracts from healthy or PMMoV-, TMGMV-, ToMV-, TMV- or ToBRFV-infected leaves. (**C**) Analytical sensitivity of the RT-LAMP primer sets MP1, MP2, and rRNA 25S. Individual Ct values for three technical replicates are shown. NTC and healthy plant RNA extract (WT) were used as negative controls. All the dilutions were made using a healthy plant RNA extract as diluent.

We also designed a LAMP test for the amplification of ToBRFV *MP* segments (Figure 4). Specific sequences were identified and used to design a pair of oligonucleotides sets named MP1 and MP2 for the exclusive and inclusive amplification of two segments of the *MP* (Figure 4A and Figure S3). As an IC, we also designed a set of oligonucleotides to detect the 25S subunit of the ribosomal RNA from solanaceous species. Figure 4B shows the results for the analytical specificity assays of MP1, MP2, and rRNA 25S oligonucleotide sets using RNA extracts from infected *N. benthamiana* (TMV), tomato (ToMV or ToBRFV), and pepper (TMGMV or PMMoV) plants. Each sample was subjected to the three tests (MP1, MP2, and 25S). An amplification signal for ToBRFV was only observed in the sample from a plant infected with this virus when using MP1 and MP2, while the ribosomal RNA was detected in all the samples, indicating that ToBRFV-negative results were due to the absence of ToBRFV rather than a failure in the testing process. Finally, we assessed the analytical sensitivity of MP1 and MP2 sets using serial dilutions of total RNA from a ToBRFV-infected tomato plant diluted in healthy plant RNA (Figure 4C). The results indicated that the MP1 set was the most sensitive, consistently detecting up to 1E-5 ng RNA/rxn, although one technical replicate of the next dilution was also positive. In contrast, the last positive dilution for the MP2 set was 1E-4 ng RNA/rxn, in general showing higher Ct values for all the dilutions as compared to MP1. In conclusion, our LAMP test exclusively detected ToBRFV, and included an IC to discard false negatives. While both primer sets worked efficiently, MP1 detected ToBRFV in an RNA dilution one order of magnitude higher than MP2.

### 3.4. Comparison of the ToBRFV Detection Methods 

To compare the new ToBRFV detection resources, we sought to generate a set of samples by mechanically inoculating tomato plants with ToBRFV and sampling them along a timecourse experiment, with three biological replicates for each data point. Our purpose was to obtain a collection of samples with diverse viral loads to interrogate our tests and assess their analytical sensitivity and repeatability using three biological replicates per data point. Protein extracts were used for the DAS-ELISA tests with MoAb 4B10 and 5A6, and RNA extracts for the RT-qPCR and LAMP tests (Table 1). As expected, the results showed that the most sensitive technique was the RT-qPCR, followed by the LAMP and the DAS-ELISA. For all these techniques, the signals obtained correlated well with the course of the infection, so for initial samples, the virus was more difficult to detect than for the later ones. In the case of the DAS-ELISA tests, ToBRFV was not detected roughly until 4 days post-inoculation (dpi). Only plant 11 (P11) gave rise to a positive signal slightly above the cut-off value for both antibodies (4B10 and 5A6), while P10 and P12 were negative at 4 dpi. Beyond this point, all of the following data points provided strongly positive readings for both immunoassays. In contrast, the nucleic acid amplification tests allowed the detection of the virus even at 1 dpi, at least for one plant. Thus, P1 showed clear positive signal with both methods, RT-qPCR and LAMP. At this time, P2 and P3 provided doubtful results, since the Ct values for the RT-qPCR test were very close to the cut-off (Ct cut-off <35) on P2, and slightly higher on P3. Neither P2 nor P3 were positive in the LAMP tests, while the IC 25S was positive, ruling out false negatives. Samples of the next data point, 2 dpi, provided more clear results for both nucleic acid amplification techniques. P4 and P5 were clearly positive with the Abiopep RT-qPCR test, but Ct values for P6 were close to the cut-off. The LAMP tests aligned well with these results, so the virus was detected in P4 and P5 by MP1 and MP2 primers but in P6, it was only barely detected with MP2 in just one technical replicate. From this time point on, for the next samples (P7-P21), the virus was readily detected by both genetic tests and Ct values decreased with the timecourse, perhaps in association with an increase in the virus titer. The results strongly suggested that the new testing methods aligned well with the likely viral load of each sample and the corresponding sensitivity of each method: Early samples with putatively very low (P1-P6) or low/intermediate viral loads (e.g., P7-P12) were only positive with the RT-qPCR and LAMP tests, although with some doubts for the second method; late samples with putative higher viral loads were positive according to all the tests, DAS-ELISA, RT-qPCR, and LAMP.

## 4. Discussion

Emergent plant viruses are a constant threat that need to be addressed quickly to avoid or limit the expansion of the new pathogens. Thus, specific testing methods that allow the identification of emergent viruses among their relatives are crucial for monitoring the progression of epidemics and implementing effective eradication measures. ToBRFV is an example of an emergent plant virus which has expanded from the Mediterranean basin to practically all the continents. Therefore, in this work, we developed an array of techniques aimed at detecting the CP and several regions of the ToBRFV genome (Figure 1): two DAS-ELISA tests against the CP, a RT-qPCR test for the *RdRP*, and a RT-LAMP test for the *MP*.

The tobamovirus virion particle is composed of thousands of CP subunits that are helicoidally arranged, wrapping the ssRNA(+), and forming a rod-shaped structure [35]. The repetition of the CP in the virion particle makes it an attractive candidate for obtaining antisera against this virus, particularly if targeting externally localized peptides. This is the most straightforward approach and the one that most of the commercial antisera follow. However, the immunization with the whole virion or CP protein yields polyclonal antisera that are likely to have cross-reactivity with other tobamoviruses, especially in the case of ToBRFV, which shares a high degree of similarity with ToMV and TMV. In contrast, MoAbs are directed against singular epitopes and are less likely to cross-react, a feature of great interest when an emergent virus is very similar to other widespread viruses. The drawback though, is that MoAbs are more difficult to obtain, and the process takes more time at a higher cost. Nevertheless, we reasoned that the extra effort and time for the development of a MoAb was justified in this case. Thus, we identified a peptide at the C-terminus of the ToBRFV CP exposed to the outer surface of the virion particle, which potentially bore enough dissimilarities with ToMV and TMV to produce specific antibodies. We used this peptide to immunize mice, and after three rounds of cloning and selection, we obtained two MoAbs which showed no cross-reactivity with the TMV CP nor with the equivalent peptides from TMV or ToMV. Next, we formulated two DAS-ELISA tests which were exclusive to ToBRFV, and which did not show cross-reactivity with TMGMV, PMMoV, ToMV, or TMV. The DAS-ELISA test formulated with the MoAb 4B10 was more sensitive than the MoAB5A6, detecting ToBRFV at least at one dilution higher. However, this difference in the analytical sensitivity was not noticed during a timecourse infection experiment, as both tests performed similarly, detecting ToBRFV after 6 dpi. From 6 to 12 dpi, the signal from both tests remained steady, perhaps indicating detection saturation. In comparison, nucleic acid amplification tests showed lower Ct values during the course of the infection, suggesting that the virus was still accumulating in the plant tissue despite the DAS-ELISA being unable to sense this increase in the viral load. 

RT-qPCR is the gold standard when discussing sensitivity in virus detection. This is especially important when the virus titer is low, as may occur in infected seed stocks [36]. The EPPO recommendation is to use the CaTa28 and CSP1325 ISF-ISHI-Veg RT-qPCRs tests. The first test targets the *MP*, whereas the second is directed to the *CP*. In addition to this, Panno et al., 2019 [24] reported a RT-qPCR test targeting the *MP* of ToBRFV. Therefore, we decided to target the *RdRP* ORF which was still untargeted. The results showed that our test could be useful for detecting ToBRFV per se, or it could be complementarily used with the aforementioned tests which target different regions of the ToBRFV genome. Under our particular conditions of reagents, equipment, and operators, the CaTa28 test was the most sensitive of either ISHI-veg tests, detecting ToBRFV in a 1E-5 ng/rxn dilution, one order of magnitude lower than CSP1325. Beyond these dilutions, the stability of the Ct measurements decreased, as reflected by some replicates which failed to amplify or produced high Ct values. In contrast, the Abiopep test continued to produce a clear signal in the rest of the dilutions, stabilizing the Ct values to around 39, which, in fact, may complicate the selection of a Ct threshold. To solve this, we collected all the Ct values coming from negative samples, including NTC, WT, and samples infected with other tobamoviruses, and observed that the lower value was close to 35. Following this cut-off value of <35, the last dilution detected by the Abiopep test was 10E-4 ng/rxn, the same as the CSP1325 test and one dilution less than the CaTa28 test. Nevertheless, it is important to remark that this threshold needs to be adjusted for each laboratory. For instance, the ISF-ISHI-Veg protocol sets a threshold at Ct <32 for the positive amplification control, whereas Botermans’ group, which followed this protocol for tracking ToBRFV outbreaks in the Netherlands, established a lower Ct threshold of <30 [21]. Importantly, aside from the in silico analysis of the primers and probe specificity, we evaluated the exclusivity of the Abiopep RT-qPCR test by using RNA extracts from plants infected with different tobamoviruses and a specific pair of primers for each one. This assay experimentally confirmed the exclusivity of our Abiopep test, which only showed amplification for ToBRFV-positive samples, while the samples infected with the other viruses were positive with their particular set of primers and probe. 

Genetic testing methods are shifting from the laboratory to the field, accelerating the decision-making process while reducing costs. To date, point-of-care testing approaches are mainly reserved for antibody tests such as immunochromatographic strips. However, these testing methods are less sensitive and specific compared with the nucleic acid amplification methods. As a result, isothermal amplification methods that do not require sophisticated equipment are gradually gaining popularity. To our knowledge, two works have been published describing LAMP tests to detect ToBRFV [26,27]. Both protocols consisted of one set of primers targeting the *RdRP* ORF, and were compared with published RT-PCR tests [14,19], confirming that the LAMP method had a higher sensitivity as had been previously demonstrated for other LAMP tests targeting different plant viruses [37,38]. Conversely, in this work, we targeted two different segments of the *MP* ORF (MP1 and MP2), adding an IC (25S) and thus facilitating the interpretation of the results. In our LAMP test, a (i) positive sample should provide a clear amplification for all three targets (MP1, MP2 and 25S), a (ii) negative sample should fail to amplify MP1 and MP2 targets while amplifying 25S, a (iii) doubtful situation would be when only one MP target (MP1 or MP2) amplified in conjunction with 25S. Examples of these outputs can be found in Table 1. Unlike Sarkes et al., 2020 [26] which tested the exclusivity using synthetic gene fragments of TMV and ToMV, Rizzo et al., 2021 [27] used plant RNA extracts obtained from plants infected with different tobamoviruses. We confirmed the exclusivity of our test following the same strategy as the second study, showing that the MP1 and MP2 sets only amplified samples infected with ToBRFV, whereas the 25S set amplified all the samples except NTC. We used the same RNA extracts from ToBRFV-infected tomato plants to evaluate the sensitivity of the Abiopep RT-qPCR test and the MP1 and MP2 LAMP tests, thus allowing the comparison between both methods. Both primer sets displayed sensitivities equal to RT-qPCR tests, with a limit of detection of 10E-5 ng/rxn for the MP1 set and 10E-4 ng/rxn for the MP2 set.

Altogether, with the experiments discussed above, we evaluated fundamental parameters such as analytical sensitivity, specificity (including inclusivity and exclusivity), repeatability and reproducibility of our tests, although, like for most analyses described in the literature, with some limitations. The analytical sensitivity was evaluated for all the tests by assaying serial dilutions of an extract from an infected plant in a healthy plant tissue extract, and also in an infection timecourse experiment. Regarding the analytical specificity, only the Spanish ToBRFV isolate was evaluated to test the inclusivity since this virus is included in the EPPO’s A2 list of quarantine pests, and access to overseas isolates is restricted. Nevertheless, the relatively short evolutionary history of ToBRFV should guarantee the inclusivity of our tests as in silico analysis of the sequences showed. To ensure the exclusivity of our tests, relevant non-target tobamoviruses which are known to infect tomato and pepper (TMV, ToMV, PMMoV, TMGMV) were tested. As happened with the inclusivity analyses, the evaluation of the alignments that we performed when designing our tests should also ensure the exclusivity of our tests. Selectivity was evaluated using tomato and *N. benthamiana* matrices with no noticeable impact on the performance of our tests. Finally, the repeatability of the tests was shown with the twenty-one plants with different viral titers that we evaluated in the timecourse experiment. Each time point was represented by three biological replicates, each one assessed by three technical replicates. 

In conclusion, we successfully achieved the goal of this work, which consisted of providing new tools for the specific and sensitive detection of ToBRFV. To our knowledge, this is the first report that gathers and describes the development, evaluation, and comparison of three testing methods with different foundations to detect ToBRFV in infected *N. benthamiana* and tomato leaves. Remarkably, these methods were compared using the same set of samples to provide clues about their selection. In the future, more tobamovirus isolates as well as matrices (e.g., pepper leaves, pepper and tomato seeds) need to be tested to expand our set of data referring the inclusivity, exclusivity, and selectivity of the tests. Additionally, more RT-qPCR amplification reagents need to be assessed to delimitate which one gives the best diagnostic performance. Meeting all standards of supra-national plant health authorities (e.g., the EPPO standards in Commission Implementing Regulation 1191/2020) will help in the generalization of the use of our methods. 

## 5. Patents

Both monoclonal antibodies reported in this manuscript are included in the Spanish Patent Application 202130416.

## Figures and Tables

**Table 1:** Comparison of ToBRFV detection methods.

		DAS-ELISA		RT-PCR	LAMP		
		MoAb 4B10	MoAb 5A6	Abiopep	MP1	MP2	rRNA 25S
1 dpi	P1	0.068 ± 0.022 ^†^	0.066 ± 0.023 ^†^	31,75 ± 0.25 ^‡^	43.45 ± 1.14 ^‡^	49.30 ± 0.86 ^‡^	23.26 ± 0.48 ^‡^
	P2	0.058 ± 0.023 ^†^	0.042 ± 0.005 ^†^	34.33 ± 0.43 ^§^	39.81 ^§^*	N.D. ^§^	19.94 ± 0.26 ^§^
	P3	0.068 ± 0.020 ^†^	0.041 ± 0.006 ^†^	35.95 ± 0.94 ^†^	N.D. ^†^	46.97 *^†^	18.36 ± 0.32 ^†^
2 dpi	P4	0.071 ± 0.012 ^†^	0.044 ± 0.003 ^†^	30.69 ± 0.13 ^‡^	39.02 ± 0.86 ^‡^	47.94 ± 1.78 ^‡^**	21.10 ± 0.24 ^‡^
	P5	0.075 ± 0.014 ^†^	0.044 ± 0.006 ^†^	29.82 ± 0.14 ^‡^	38.05 ± 1.68 ^‡^	47.81 ± 2.31 ^‡^**	18.93 ± 0.01 ^‡^
	P6	0.068 ± 0.015 ^†^	0.039 ± 0.003 ^†^	34.78 ± 0.46 ^§^	N.D. ^§^	49.74 ^§^*	22.17 ± 0.48 ^§^
3 dpi	P7	0.068 ± 0.028 ^†^	0.042 ± 0.008 ^†^	30.30 ± 0.62 ^‡^	39.19 ± 0.81 ^‡^	47.81 ± 0.80 ^‡^	18.53 ± 0.22 ^‡^
	P8	0.062 ± 0.020 ^†^	0.045 ± 0.003 ^†^	25.93 ± 0.13 ^‡^	33.67 ± 0.76 ^‡^	43.18 ± 1.18 ^‡^	18.81 ± 0.17 ^‡^
	P9	0.051 ± 0.028 ^†^	0.052 ± 0.023 ^†^	26.79 ± 0.02 ^‡^	35.71 ± 1.00 ^‡^	45.53 ± 2.76 ^‡^	18.10 ± 0.16 ^‡^
4 dpi	P10	0.09 ± 0.01 ^†^	0.1 ± 0.004 ^†^	22.34 ± 0.07 ^‡^	22.60 ± 0.24 ^‡^	31.57 ± 0.43 ^‡^	15.02 ± 0.41 ^‡^
	P11	0.18 ± 0.01 ^§^	0.22 ± 0.04 ^§^	10.35 ± 0.03 ^‡^	21.86 ± 0.21 ^‡^	30.63 ± 0.063 ^‡^	17.23 ± 0.79 ^‡^
	P12	0.07 ± 0.01 ^†^	0.10 ± 0.02 ^†^	10.04 ± 0.14 ^‡^	22.67 ± 0.2 ^‡^	32.64 ± 1.16 ^‡^	16.24 ± 0.36 ^‡^
6 dpi	P13	2.49 ± 0.11 ^‡^	3.34 ± 0.02 ^‡^	11.37 ± 0.48 ^‡^	13.23 ± 1.22 ^‡^	20.81 ± 0.47 ^‡^	16.14 ± 0.15 ^‡^
	P14	2.46 ± 0.18 ^‡^	3.38 ± 0.04 ^‡^	21.63 ± 0.07 ^‡^	12.08 ± 0.6 ^‡^	19.8 ± 0.30 ^‡^	16.23 ± 0.29 ^‡^
	P15	2.41 ± 0.08 ^‡^	3.26 ± 0.02 ^‡^	22.79 ± 0.12 ^‡^	13.77 ± 0.97 ^‡^	20.86 ± 0.19 ^‡^	17.3 ± 0.48 ^‡^
8 dpi	P16	2.54 ± 0.2 ^‡^	3.34 ± 0.05 ^‡^	12.96 ± 0.53 ^‡^	11 ± 0.64 ^‡^	19.89 ± 0.21 ^‡^	17.02 ± 3.33 ^‡^
	P17	2.52 ± 0.28 ^‡^	3.42 ± 0.12 ^‡^	12.00 ± 0.67 ^‡^	10.04 ± 0.28 ^‡^	17.78 ± 0.77 ^‡^	19.72 ± 3.16 ^‡^
	P18	2.21 ± 0.56 ^‡^	3.20 ± 0.05 ^‡^	13.22 ± 0.08 ^‡^	10.46 ± 0.22 ^‡^	18.12 ± 1.15 ^‡^	17.80 ± 0.11 ^‡^
12 dpi	P19	2.49 ± 0.06 ^‡^	3.38 ± 0.01 ^‡^	13.29 ± 0.06 ^‡^	10.42 ± 0.41 ^‡^	17.30 ± 0.19 ^‡^	17.57 ± 0.28 ^‡^
	P20	2.34 ± 0.2 ^‡^	3.32 ± 0.02 ^‡^	10.83 ± 0.12 ^‡^	10.39 ± 0.62 ^‡^	17.66 ± 0.30 ^‡^	18.92 ± 1.20 ^‡^
	P21	2.53 ± 0.28 ^‡^	3.35 ± 0.03 ^‡^	10.15 ± 0.03 ^‡^	11.23 ± 0.19 ^‡^	18.61 ± 0.29 ^‡^	19.15 ± 1.30 ^‡^

Twenty-one tomato plants were infected with ToBRFV and systemic leaves were individually sampled into subgroups of three plants at 1, 2, 3, 4, 6, 8, and 12 days post-inoculation (dpi). Samples were analyzed using the DAS-ELISA, RT-qPCR, and LAMP tests described in the text. Columns represent the detection methods and rows the three plants analyzed per time point. Data are shown as the mean ± S.D. of three technical replicates. Symbols: ^†^ Negative result; ^‡^ Positive result; ^§^ Doubtful result; * One technical replicate; ** Two technical replicates.

## Data Availability

Not applicable.

## Supplementary Materials

The following are available online at https://www.mdpi.com/article/10.3390/v13091680/s1, Figure S1: Antisera immunogenicity characterization and hybridoma production and selection; Figure S2: Estimation of the Abiopep RT-qPCR test threshold; Figure S3: Evaluation of the analytical inclusivity of the tests. Table S1: Detailed Ct values of the RT-qPCR tests shown in Figure 3; Table S2: Oligonucleotides used in this study.
