# Supplementary material for: New Resources for the Specific and Sensitive Detection of the Emerging Tomato Brown Rugose Fruit Virus
Source: Viruses. 2021 Aug 25;13(9):1680. doi: 10.3390/v13091680 (PMC8473139; doi:10.3390/v13091680)
Supplement: Supplementary file 1 [file viruses-13-01680-s001.zip › viruses-1304013-supplementary.pdf]

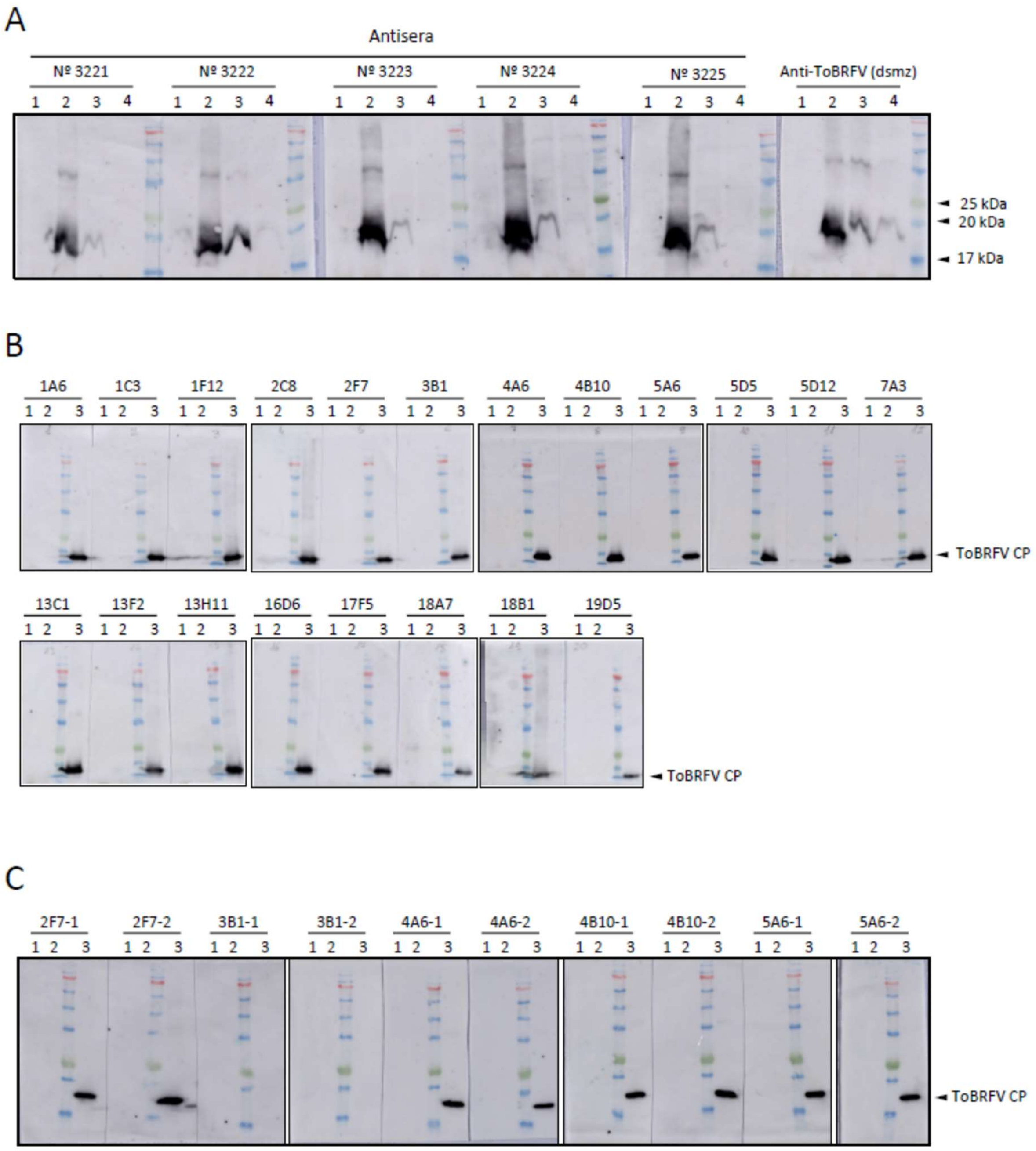

Figure S1: Antisera immunogenicity characterization and hybridoma production and selection. A) Western blots to check the reactivity of five antisera (3221, 3222, 3223, 3224 and 3225) from five mice immunized with the selected ToBRFV CP peptide. The antisera and commercial ToBRFV antibody (DSMZ) were challenged against extracts from healthy plant (lane 1) and tomato plants infected with ToBRFV (lane 2), TMV (lane 3) or ToMV (lane 4). **B)** Western blots to check the reactivity of 20 hybridoma cell lines. Lanes 1 of each blot were loaded with healthy plant extract, lanes 2 with an extract from tomato plants infected with TMV, and lanes 3 with a 1:100 diluted extract from tomato plants infected with ToBRFV. **C)** Western blots to check the reactivity of 10 hybridoma subclone culture supernatants from five parental clones (2F7, 3B1, 4A6, 4B10 and 5A6) to ToBRFV-infected tomato leaf extracts in Western blot. Lanes 1 of each blot were loaded with healthy plant extract, lanes 2 with extract from tomato plants infected with TMV, and lanes 3 with a 1:100 diluted extract from tomato plants infected with ToBRFV. The subclones chosen for the monoclonal antibody production were 4B10-1 and 5A6-1.

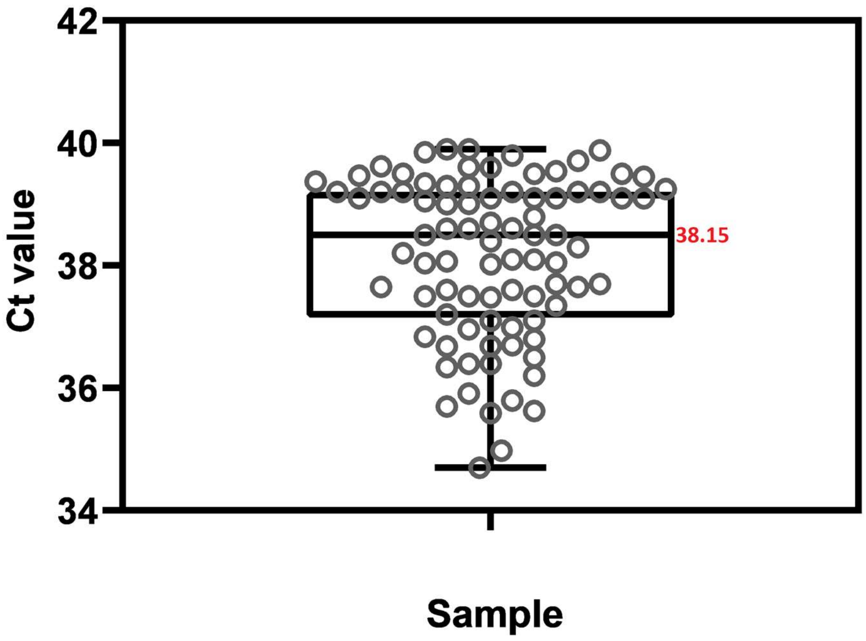

Figure S2: Estimation of the Abiopep RT-qPCR test threshold. Meta-analysis gathering all the negative samples analyzed by Abiopep´s diagnostic service in 2020, using the Abiopep RT-qPCR test. The plot contains 85 values and the Ct median resulted in 38.15 and the lower value in 34.7.

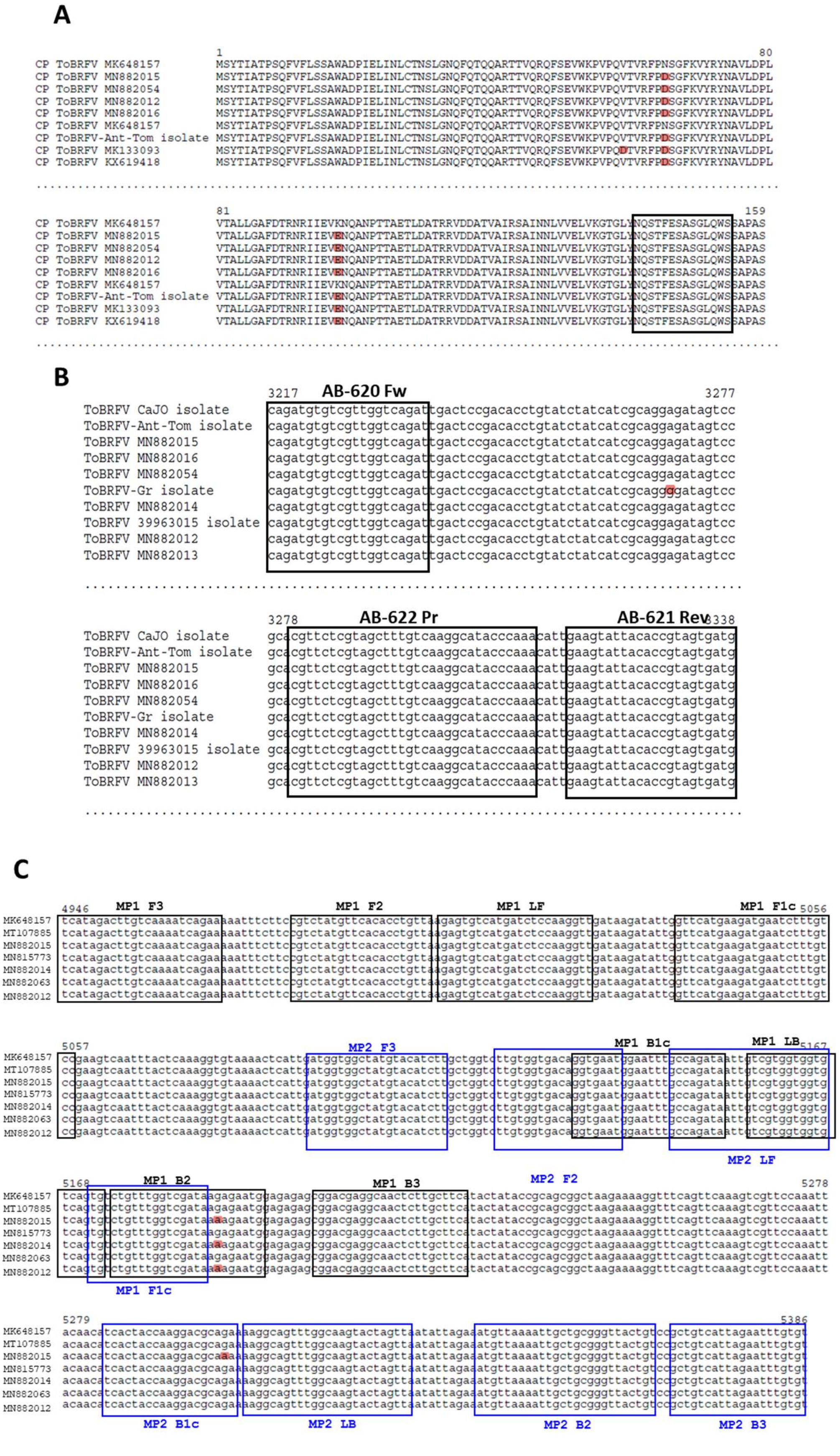

Figure S3: evaluation of the analytical inclusivity of the tests. **A)** MoAbs recognition epitope. **B)**, Abiopep's RT-qPCR test oligonucleotides. **C)** MP1 and MP2 LAMP primers. All the sequences shown are from different ToBRF isolates extracted from NCBI. Alignments were performed using Benchling webtool.

Table S1: detailed Ct values of the RT-qPCR tests shown in Figure 3.

RNA extract concentration (ng/µl)	Mean Ct			SD
	R1	R2	R3	
**Abiopep Primers**				
10	14.96	14.70	14.96	0.15
1	18.36	18.39	18.31	0.04
1,00E-01	21.96	21.88	21.87	0.05
1,00E-02	25.36	25.37	25.50	0.08
1,00E-03	29.25	28.90	28.91	0.20
1,00E-04	32.36	32.49	32.28	0.10
1,00E-05	37.42	35.82	36.07	0.86
1,00E-06	39.35	37.72	39.31	0.93
1,00E-07	39.14	36.89	38.81	1.22
WT	39.98	N.D		
**Enza Zaden Primers**				
10	14.35	14.77	14.40	0.23
1	17.55	17.72	17.63	0.09
1,00E-01	20.85	20.98	20.86	0.08
1,00E-02	24.35	24.24	24.28	0.06
1,00E-03	27.64	27.54	27.48	0.08
1,00E-04	30.87	31.20	30.91	0.18
1,00E-05	33.88	34.43	33.79	0.35
1,00E-06	N.D	36.96	N.D	
1,00E-07	36.98	36.99	N.D	
WT	N.D	35.92		
**CSP Labs Primers**				
10	17.42	17.50	17.49	0.04
1	20.75	20.66	20.66	0.05
1,00E-01	23.95	24.12	20.08	0.09
1,00E-02	27.39	27.33	27.34	0.03
1,00E-03	30.60	30.78	30.49	0.14
1,00E-04	34.28	35.14	33.62	0.76
1,00E-05	36.14	N.D	35.96	0.13
1,00E-06	N.D	N.D	N.D	
1,00E-07	N.D	35.94	37.02	
WT	36.94	N.D		

Ct values from the comparison of the CaTa28, CSP1325 and Abiopep RT-qPCR tests. The Ct value for each technical replicate (R1-R3) and RNA dilution are tabulated together with the standard deviation (SD).

Table S2: oligonucleotides used in this study.

Oligonucleotide	Sequence (5'-3')	Notes
**F3 MP1**	TCATAGACTTGTCAAAATCAGAA	RT-LAMP MP1
**B3 MP1**	GAAGCAAGAGTTGCCTCG	
**FIP MP1**	GGACAAAGATTCATCTTCATGAACCCGTCTATGTTCACACCTGTT	
**BIP MP1**	AGGTGAATGGAATTTGCCAGATCATTCTCTTATCGACCAAACAG	
**LF MP1**	ACCTTGGAGATCATGACACTCT	
**LB MP1**	TGTCGTGGTGGTGTCAGTG	
**F3 MP2**	GATGGTGGCTATGTACATCT	RT-LAMP MP2
**B3 MP2**	ACACAAATTCTAATGACAGCG	
**FIP MP2**	TATCGACCAAACAGACACTGACACTTGTGGTGACAGGTGAA	
**BIP MP2**	TCACTACCAAGGACGCAGAACAGTAACCCGCAGCAATT	
**LF MP2**	CACCACGACAATTATCTGGCA	
**LB MP2**	AAGGCAGTTTGGCAAGTACTAGTT	
**F3 25S**	AGCCAAGCGTTCATAGCG	RT-LAMP rRNA25S
**B3 25S**	GCTTCTAGCCCGGATTCTGA	
**FIP 25S**	CACGTTCCCTATTGGTGGGTGAATCCTTCGATGTCGGCTCTT	
**BIP 25S**	CACACAATTGGCCATCGCGCAGTCATAATCCAGCACACGG	
**LF 25S**	ACTTGGTGAATTCTGCTTCACA	
**LB 25S**	AAGCCAGTGGCGCGAAG	
**CaTa28 Fwd.**	GGTGGTGTCAGTGTCTGTTT	RT-qPCR ISHI veg/ENZA ZADEN ToBRFV primers
**Cata28 Rev.**	GCGTCCTTGGTAGTGATGTT	
**CaTa28 Pr.**	/56-FAM/AGAGAATGGAG/ZEN/AGAGCGGACGAGG/3IABKFQ/	
**CSP1325 Fwd.**	CATTTGAAAGTGCATCCGGTTT	RT-qPCR ISHI veg/CSP Labs ToBRFV primers
**CSP1325 Rev.**	GTACCACGTGTGTTTGCAGACA	
**CSP1325 Pr.**	/56-FAM/ATGGTCCTCTGC/ZEN/ACCTGCATCTTGAGA/3IABKFQ/	
**AB-620**	CAGATGTGTCGTTGGTCAGAT	RT-qPCR Abiopep ToBRFV test
**AB-621**	CATCACTACGGTGTAATACTTC	
**AB-622**	/56-FAM/CGTAGCTTTGTCA/ZEN/AGGCATACCCAAA/3IABKFQ/	
**AB-233**	AGCAGTTCTCTGAYGTGTGGA	RT-qPCR PMMoV test
**AB-234**	AGAAGTGCCGACACTAGWGAA	
**AB-235**	/56-FAM/CGACCGCTACAGTTAGATTTCCTGCTAC/3IABKFQ/	
**AB-236**	GTAGCTATAAGGGCTTCAATCA	RT-qPCR TMGMV test
**AB-237**	TGGTCCARACAAGTCCACTA	
**AB-238**	/56-FAM/TGAACTGGTTCGTGGAACTGGCAT/3IABKFQ/	
**AB-753**	CTCGCAAAGTTTCGAACCAA	RT-qPCR ToMV test
**AB-754**	GACGTCTCGGCTTCATCT	
**AB-755**	/56-FAM/GTTCAGGCGGA/ZEN/AGGCCTAAACCAA/3IABKFQ/	
**AB-756**	GTTGATGAGTTCATGGAAGATGT	RT-qPCR TMV test
**AB-757**	ATTTCTATAGTTCTTGTTCGGC	
**AB-758**	/56-FAM/CGATCTCGAACCGGAA/ZEN/AAAAGAGTGA/3IABKFQ/	
